# Coloclyster of Red Peony Root Granules Alleviates Moderately Severe Acute Pancreatitis: A Double-Blinded, Placebo-Controlled, Randomized Clinical Trial

**DOI:** 10.1155/2020/8401239

**Published:** 2020-07-22

**Authors:** Fangyong Yang, Xiuzhong Qi, Yiqi Du, Yan Chen, Meitang Wang, Haitao Huang, Dezeng Zhu, Xiaoqiang Yue, Lina Wang

**Affiliations:** ^1^Department of Traditional Chinese Medicine, Changhai Hospital, Naval Medical University, No. 168 Changhai Road, Shanghai 200433, China; ^2^Department of Integrative Medicine, Huashan Hospital, Fudan University, No. 12 Middle Urumqi Road, Shanghai 200040, China; ^3^Department of Traditional Chinese Medicine, Qingdao Special Service Sanatorium of PLA Navy, No. 27 Xianggang West Road, Qingdao 266071, China; ^4^Department of Gastroenterology, Changhai Hospital, Naval Medical University, No. 168 Changhai Road, Shanghai 200433, China; ^5^Department of Emergency, Changhai Hospital, Naval Medical University, No. 168 Changhai Road, Shanghai 200433, China; ^6^Department of Critical Care Medicine, Laixi People's Hospital, No. 69 Yantai Road, Laixi, 266600, China; ^7^Department of Traditional Chinese Medicine, Changzheng Hospital, Naval Medical University, No. 415 Fengyang Road, Shanghai 200003, China

## Abstract

The red peony root derived from *Paeonia lactiflora* has been applied to treat human inflammatory diseases. To investigate its therapeutic potential in treating moderately severe acute pancreatitis (MSAP), which has been rarely studied, this study was designed as a double-blinded, placebo-controlled, randomized clinical trial. A total of 60 MSAP patients were enrolled and randomly divided into an experimental (*n* = 30) group and a control group (*n* = 30), who received a coloclyster of 15 g of red peony root or placebo granules dissolved in 150 mL of water, respectively. The patients' demographic and clinical characteristics were recorded. The results showed that the experimental group had a shorter remission time of fever (*p* < 0.05) and abdominal pain (*p* < 0.01) and faster resumption of self-defecation (*p* < 0.01) than did the control group. In addition, the coloclyster of red peony root decreased the modified Balthazar CT score as well as the serum interleukin-6 and tumor necrosis factor-alpha levels to a greater extent than did the placebo coloclyster (*p* < 0.05). The remission times for the normalization of white blood cells and percentage of neutrophils and lymphocytes in the experimental group were also significantly shorter than those in the control group (*p* < 0.05). In conclusion, a coloclyster of red peony root could help alleviate the clinical symptoms and shorten the course of MSAP by possibly attenuating systematic inflammation. This trial is registered with 14004664.

## 1. Introduction

Acute pancreatitis (AP) is one of the most common causes of acute abdominal pain in clinical practice, which is characterized by inflammation and autodigestion of the pancreas [1]. Most AP are often self-limiting. However, about 20% of cases are clinically severe [2]. Globally, in-hospital mortality rate of patients with severe acute pancreatitis (SAP) are estimated to be 5%–42% [3–6]. Patients with SAP usually present with fever, abdominal pain, subcutaneous bleeding, sepsis, and sometimes systematic inflammatory response syndrome and multiple organ dysfunction syndrome. In recent years, the therapeutic potential of multidisciplinary treatment of SAP has been widely acknowledged and recommended [7], especially the introduction of traditional Chinese medicine. A great deal of evidence has demonstrated that a variety of traditional Chinese herbs exhibit anti-inflammatory activity [8, 9]; in particular, the red peony root derived from *Paeonialactiflor* is expected to be of special clinical significance in the treatment of SAP.

Our previous study has already shown that a red peony root-based therapeutic regimen can successfully alleviate the symptoms of SAP patients [10]. The main active component in red peony root is glucosides [11], which are able to improve blood circulation [12, 13] and gastrointestinal function along with exhibiting anti-inflammatory, antioxidant, and anti-endotoxin activities [14–19]. However, these pharmacological activities and the potential molecular mechanism of red peony root are not completely understood in moderately severe acute pancreatitis (MSAP). Thus, based on our pilot study, we conducted this double-blinded, placebo-controlled, randomized clinical trial, aiming at further investigating the efficacy, feasibility, and safety of a coloclyster of red peony root in the management of patients with MSAP. These results might provide new evidence for optimization of the current treatment for AP patients.

## 2. Materials and Methods

### 2.1. Study Design

**Figure 1:**
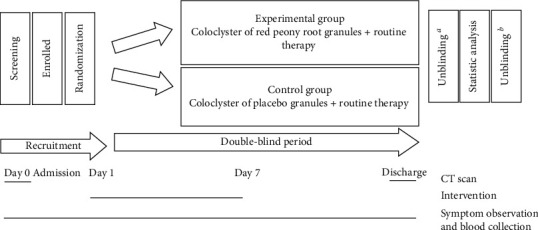
Study design, intervention: 15 g of red peony root or placebo granules dissolved in 150 ml of water at 35°C–40°C at 9:00 am and 3:00 pm, twice a day for 7 days; routine therapy: pantoprazole sodium, gabexate mesylate, ulinastatin, cefoperazone sodium, and alprostadil.

This was a randomized, double-blind study comprising a screening period and a double-blind treatment period (Figure 1). The primary objective of the study was to evaluate the optimal efficiency of a coloclyster of red peony root to routinely treat patients with MSAP. As a secondary objective, the patients' systematic inflammation level was also assessed.

This study was approved by the institutional research and the Ethics Committee of Changhai Hospital, and all experiments were performed in accordance with relevant guidelines and regulations. The study was registered in the Chinese Clinical Trial Registry (http://apps.who.int/trialsearch/Default.aspx) on May 19, 2014. The registration number of this clinical trial was CHICTR-TRC-14004664.

### 2.2. Inclusion/Exclusion Criteria

Patients aged 18–70 years old and diagnosed with MSAP in the Department of Gastroenterology and Emergency of Changhai Hospital from June 2014 to March 2015 were eligible for this study. The main symptoms in all the age patients are abdominal pain and self-defecation.

The diagnostic criteria were in accordance with the global initiative for MSAP [7] on the basis of the diagnostic criteria for AP, including one of the following features during the acute phase: (1) Ranson's score ≥3, (2) Acute Physiology and Chronic Health Evaluation II (APACHE II) score >8, (3) Bedside Index for Severity in Acute Pancreatitis (BISAP) score ≥3, (4) Modified Computed Tomography Severity Index (MCTSI) score ≥4, and (5) transient (<48 h) organ dysfunction. These included pseudocysts, pancreatic fistulas, or pancreatic abscesses that required intervention during the recovery period. Informed consent was obtained from all participants. Key exclusion criteria included a disease course lasting more than 72 h after admission, pregnancy, a previous history of AP, severe primary comorbidities including cardiopulmonary or hematological disorders, shock, disseminated intravascular coagulation, acute respiratory distress syndrome, being treated with Chinese herbs before admission, or enrollment in another clinical trial.

### 2.3. Randomization and Blinding

The sample size and power calculations were based on the superiority test. The sample size was calculated based on *α* = 0.05 and (1 − *β*) = 80%, as a previous paper described [10]. The “lost to follow-up rate” was anticipated to be 10%, and a final sample of 30 in each group was determined. An independent statistician generated sixty random numbers using PROC PLAN of SAS 9.2 (SAS Institute Inc., Cary, NC, USA) and kept a sealed copy of the number list. The pharmacy department staff labeled the red peony root granules and the placebo with the randomized numbers. To maintain the study as blinded, there were no discernible differences between the red peony root granules and the placebo granules. The herbs and the placebo were subsequently distributed to each investigator. The randomization was controlled by an investigational center, who transmitted the randomization form containing basic information for each enrolled subject by facsimile to the statistician. Then, the statistician returned the randomization form filled in with the established random number (a specified ID number) to the investigational center. After this process, the investigational center provided the specified ID number to the investigators and the clinical pharmacists distributed the red peony root granules or placebo granules according to the corresponding number. The randomization allocation ratio at the sites was 1 : 1. All participants, investigators, investigational center, and pharmacists were blinded to the treatment. All procedures involving randomization and blinding were audited by the Ethics Committee of Changhai Hospital.

The key stopping rules for individuals included the occurrence of complications or special physiological changes that rendered them not suitable to continue, poor compliance that could affect the safety and efficacy evaluation, occurrence of adverse or serious adverse events for which it was not appropriate for patients to continue, refusal to continue because of personal reasons put forward by patients, and decisions to withdraw made by researchers for other circumstances.

### 2.4. Description of the Drug

The red peony roots were purchased from the Jiangyin Tianjiang Pharmaceutical Company (Jiangsu, China) and were identified by Professor Hailiang Xin (Navy Military Medical University, Shanghai, China). Granular samples of 0.05 g were accurately weighted into a 50 mL volumetric flask and sonicated in 70% alcohol using an ultrasonicator for 30 min at room temperature. Then, 1.0 mL of the extraction was transferred to a 1.5 mL microcentrifuge tube and centrifuged for 10 min at 13,000 rpm. The supernatant was collected and stored at 4°C pending analysis.

The HPLC-MS analysis was performed using an Agilent 1290 Infinity equipped with an Agilent 6538 UHD and Accurate-Mass Q-TOF LC/MS. Samples (4 *μ*L) were separated on a Waters XBridge HSS T3 column (2.1 mm × 100 mm, i.d. 2.5 *μ*m) using a gradient elution. The mobile phase consisted of two solvents: solvent A was 0.1% formic acid and solvent B was a mixture of 0.1% formic acid in acetonitrile. The gradient elution was performed as follows: 0–2 min, 5% B; 2–10 min, 5–20% B; 10–14 min, 20% B; 14–17 min, 20–95% B; 17–20 min, and 95% B. The flow rate was 0.4 mL/min, and the column temperature was 25°C. The MS identification was operated using an electrospray ionization (ESI) source in positive and negative ion modes. The ionization source conditions (positive/negative ion mode) were as follows: nebulizer pressure of 50 psig; drying gas flow rate of 11 L/min; drying gas temperature of 350°C; capillary voltage of 4.0 kV/−3.5 kV; and fragmentor voltage of 120 V. The spectrometric data were collected from *m*/*z* 500 to 1500 Da in positive and negative ion modes and stored in the centroid mode.

### 2.5. Interventions

All patients enrolled in this study were randomly divided into one of two groups by the methods described above: the experimental group and the control group. Patients received a coloclyster of 15 g of red peony root (experimental group) or placebo granules (control group) (Jiangyin Tianjiang, China) dissolved in 150 mL of water at 35°C–40°C at 9:00 am and 3:00 pm, twice a day for 7 days. The dosages were equal for all the age groups. In addition, all patients in the two groups had fasted, received gastrointestinal decompression, antacids, and antibiotics, and were supported by parenteral alimentation. A dosage of 20 mg of pantoprazole sodium (H20052022, Liaoning Nirvana Pharmaceutical, China) q12h, 300 mg of gabexate mesylate (H20059767, Changzhou Siyao Pharm, China) qd, 300,000 U of ulinastatin (H19990134, Techipool, China) qd, 1.5 g of cefoperazone sodium/sulbactam sodium (H20020597, Pfizer, China) bid, and 20 *μ*g of alprostadil (H20094203, Harbin Pharmaceutical, China) qd were administered to both groups. Early enteral nutrition was also necessary. A dosage of 125 g of Short-Peptide Enteral Nutrition Powder (H20170170, Milupa GmbH, Germany) qd was administered to both groups at the beginning of treatment and was replaced by 1500–2000 ml of Enteral Nutritional Emulsion (TPF-D) (H20140192, Fresenius Kabi Deutschland GmbH, Germany) qd after 3–5 d.

### 2.6. Outcome Measurements

The vital signs, clinical symptoms of abdominal pain and self-defecation, and adverse events were recorded at 6:30 am and 4:30 pm every day in all patients to analyze the therapeutic efficacy of the treatment. The complete remission time of abdominal pain and onset of self-defecation were the primary outcomes used to determine the overall effect (an observation recording form which was designed referring to the APACHE II and Modified Balthazar CT score systems was provided as an appendix). Secondary outcomes were measured as the remission of fever, the modified Balthazar CT score, the remission times for the normalization of white blood cells, and the percentage of neutrophils and lymphocytes. The levels of serum interleukin-6 (IL-6) and tumor necrosis factor-alpha (TNF-*α*), and hospital stay and cost of hospitalization were also measured or calculated Table 1.

Peripheral venous samples were collected from all patients at admission and at 6:30 am and 4:30 pm every day during the treatment. The nurses who were responsible for the different patients collected the samples. The serum amylase, C-reactive protein (CRP), IL-6, and TNF-*α* levels were detected, and routine blood tests were conducted by the clinical laboratory of Changhai hospital. Computed tomography (CT) scans were performed at admission and whenever necessary during the treatment. Patients were allowed to be discharged when they satisfied all of the following conditions, including the disappearance of clinical symptoms, the normalization of blood tests, a reduced exudation as shown by CT examination, or the formation of a pseudocyst. The modified Balthazar CT score was obtained by the same person for all patients. Adverse events were observed and recorded for all participants through the period by the investigator.

### 2.7. Statistical Analysis

All statistical analyses were conducted by using SPSS 20.0 software (SPSS Inc., Chicago, IL, USA) by an independent statistician. The superiority test was defined as the lower limit of the two-sided 95% confidence interval (CI). All analyses were based on the intention-to-treat (ITT) principle, but per-protocol (PP) was used in some cases. The categorical data are presented as percentages (%) and compared by the chi-squared test. The continuous data are presented as the mean ± standard deviation ($\bar{x}\pm s$), and the differences between the two groups were tested by the independent *t* test or the rank-sum test, when applicable. The differences of continuous variables before and after treatment within each group were compared by the paired *t* test. A *p* value less than 0.05 was considered to be statistically significant.

## 3. Results

### 3.1. Identification of the Red Peony Root

**Figure 2:**
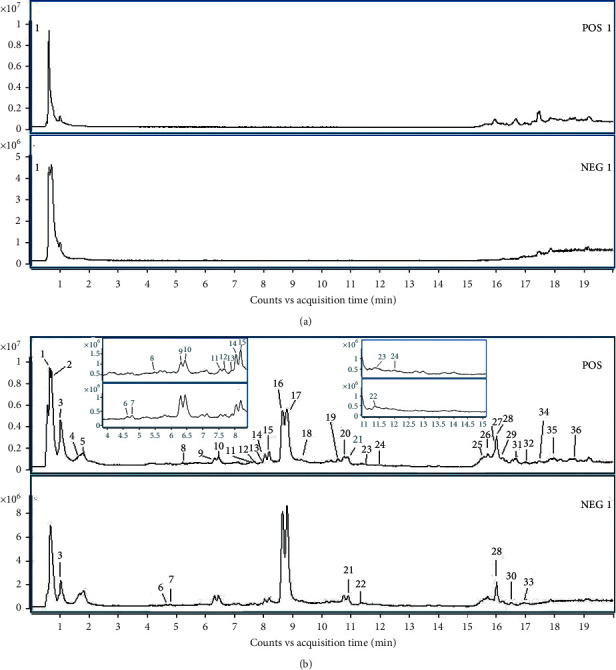
The TICs in the positive ion mode and the negative ion mode of the control (a) and the red peony root (b).

The probable chemical constituents of the red peony root were collected according to the related literature that previously reported the constituents in the red peony root and related species. A database of the known chemical constituents was established using the “Formula-Database-Generator” software and then introduced into the “Masshunter Qualitative Analysis” software system provided by Agilent. The identification of the red peony root was carried out by retrieving the MS data of the samples in the database. The molecular ion peaks of [M + H]^+^, [M + Na]^+^, and [2M + H]^+^ in the positive ion mode and [M-H]^−^, [M + HCOO]^−^, and [2M-H]^−^ in the negative ion mode were searched. The mass ranges were set at *m*/*z* 50–1500 Da, and the mass error of the predicted chemical formula should be less than 5.00 ppm. Finally, a total of 36 compounds were identified in the positive ion mode and negative ion mode in the red peony root (Table 2). The total ion chromatograms (TICs) in positive ion mode and negative ion mode of the control and the red peony root are shown in Figure 2.

### 3.2. Demographic and Clinical Characteristics

A total of 77 adult patients aged 18–70 years old diagnosed with MSAP were admitted to the Department of Gastroenterology and Emergency of Changhai Hospital from June 2014 to March 2015 and were eligible for this study. There were 17 patients who were excluded due to the presence of a previous history of AP, pregnancy, severe primary comorbidities including cardiopulmonary or hematological disorders, shock, disseminated intravascular coagulation, acute respiratory distress syndrome, a disease course lasting more than 72 h upon admission, or being treated with Chinese herbs before admission.

**Figure 3:**
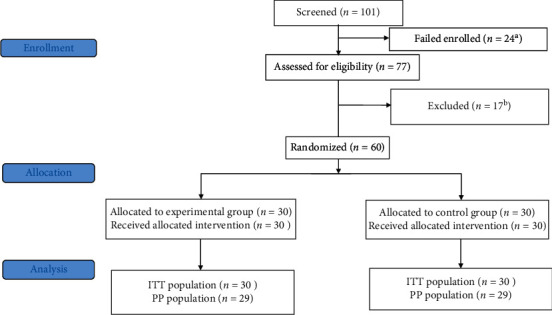
Patient flow chart. ITT: intent-to-treat; PP: per protocol. ^*a*^Inclusion criteria not met, *n* = 24; ^*b*^exclusion criteria met, *n* = 17 (pregnancy, *n* = 3; disease course lasting more than 72 h upon admission, *n* = 6; severe primary comorbidities, *n* = 5; older than 70, *n* = 1; declined the coloclyster, *n* = 1; coloclyster administered in another hospital, *n* = 1).

The remaining 60 MSAP patients were randomly divided into an experimental (*n* = 30) and a control group (*n* = 30), and there were no losses or exclusions after randomization. The demographic and clinical characteristics of the patients were retrieved from the computerized database (Figure 3). The mean ages of the patients in the experimental and control groups were 49.5 ± 13.6 and 47.2 ± 13.6 years, respectively. Biliary diseases and alcohol consumption were the common causes of MSAP in both groups. The average APACHE II scores were 9.1 ± 2.2 and 8.4 ± 2.3 in the experimental and control groups, and their mean modified Balthazar CT scores were 4.1 ± 0.4 and 4.3 ± 0.7, respectively. No significant differences in terms of sex, age, body mass index, etiology of MSAP, APACHE II score, or modified Balthazar CT score were found between the two groups (*p* > 0.05) (Table 3).

### 3.3. Therapeutic Efficacy of the Coloclyster of the Red Peony Root Granules

**Figure 4:**
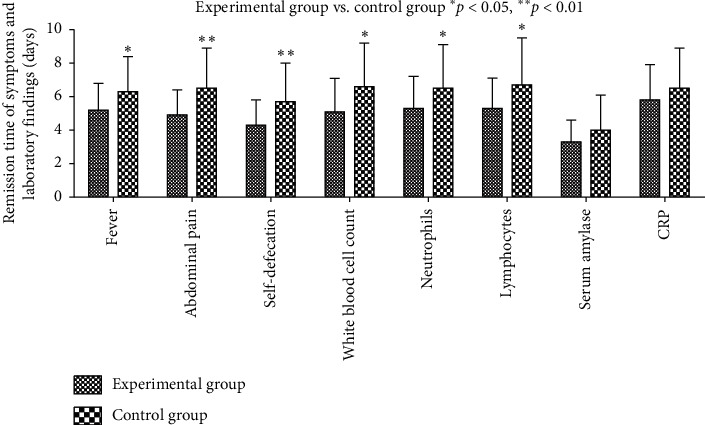
Remission time of different symptoms and laboratory findings in the two groups (*n* = 30). Symptoms were as indicated on the *X* axis.

The disease severity was investigated by evaluating the clinical symptoms, laboratory tests, and radiological results before and after treatment (Figure 4). For each group, the number of participants included in each analysis was 30, and the analysis was according to the original assigned groups.

**Figure 5:**
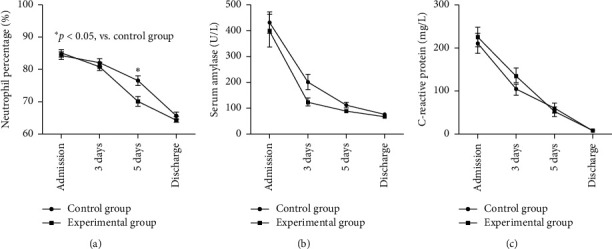
Changes of neutrophil percentage (a), amylase (b), and CRP level (c) at admission, 3 and 5 days after treatment and before discharge between the two groups.

Compared with the control group, the patients in the experimental group had a shorter remission time for fever and abdominal pain and a faster onset of self-defecation (5.2 ± 1.6 vs. 6.3 ± 2.1, *p*=0.040; 4.9 ± 1.5 vs. 6.5 ± 2.4, *p*=0.005; 4.3 ± 1.5 vs. 5.7 ± 2.3, *p*=0.001) (Figure 4). The normalization of the white blood cell count and percentages of neutrophils and lymphocytes took a shorter time in the experimental group compared to the control group (*p* < 0.05) (Figure 5(a)). The amylase levels in the experimental group also had a more obvious downward tendency (Figure 5(b)). At admission, the patients' amylase and CRP levels were obviously increased, and they decreased to the normal range within 3–6 days after treatment (Figure 5(c)).

Before treatment, there were no significant differences in serum IL-6 (*p*=0.200) or TNF-*α* (*p*=0.155) levels between the two groups. After treatment, the IL-6 and TNF-*α* levels were significantly decreased in the experimental group (*p* < 0.01). Furthermore, the coloclyster of the red peony root was more effective at decreasing the IL-6 level (33.0 ± 15.0 vs. 15.3 ± 20.4, *p*=0.012), and a similar trend for the TNF-*α* level was observed (106.7 ± 72.5 vs. 43.3 ± 83.6, *p*=0.035) (Table 4).

The modified Balthazar CT score was introduced to evaluate the disease severity, which was comparable between the two groups (*p*=0.165). The coloclyster of red peony root decreased the modified Balthazar CT score to a greater extent than that of the placebo (*p*=0.015) (Table 5).

### 3.4. Hospitalization and Cost

As shown in Table 6, the patients in the experimental group had a shorter hospital stay (11.3 ± 2.3 days vs. 13.8 ± 2.7 days, *p* ≤ 0.001) and a lower cost of hospitalization (4.15 ± 0.64 × 10,000 RMB vs. 4.84 ± 0.88 × 10,000 RMB, *p*=0.001), compared with those in the control group, indicating that the administration of the traditional Chinese medicine of red peony root granules for the treatment of MSAP could help the patients recover faster and reduce their economic burden.

### 3.5. Feasibility and Safety

All of the patients could tolerate the coloclyster procedure and the treatment success rate was 100%. In the experimental group, one patient had diarrhea and one patient had abdominal distension and pain, while in the placebo group, two patients had abdominal distension and pain and one patient had abdominal distension, nausea, and vomiting. The incidence rates of the adverse events in the two groups were not statistically significant (6.67% vs. 10.0%, *p* > 0.05) and were associated with the primary disease, the high location, and the rapid administration of the coloclyster. All of the adverse events were cured by conservative treatment.

## 4. Discussion

Currently, due to a less healthy lifestyle, the incidence of pancreatic diseases is on the rise in China. Patients with AP may progress rapidly into septic shock, which is associated with a very poor outcome. Thus, the development of effective treatments for AP is vital to the improvement of the clinical outcome in such patients. Our study identified a new comprehensive treatment strategy that introduced the application of traditional Chinese medicine in the management of MSAP patients, which may deepen the current understanding of the therapeutic potential of traditional Chinese medicine in treating human diseases.

So far, the pathophysiology of SAP has been extensively studied, and four hypotheses have been widely acknowledged, including (1) pancreatic autodigestion, (2) cascade inflammatory response induced by cytokines from activated inflammatory cells, (3) pancreatic microcirculation dysfunction, and (4) translocation of gut microbiota to the pancreas [7, 18]. The preactivation of amylase in the pancreas plays an important role in the pathogenesis of AP and can be used as an independent prognostic factor [18]. In our cohort, all patients had increased amylase levels and there was a more obvious downward tendency of the amylase level in the experimental group after treatment, indicating that the red peony root therapy was probably able to restore the impaired structure and function of the pancreatic tissue.

Previous experimental evidence has also demonstrated that red peony root extracts can reduce the serum amylase level in rats [20] in addition to other inflammatory mediators [21], thus contributing to the alleviation of inflammation. In addition, it is well acknowledged that the cascade reaction induced by inflammatory cells and cytokines regulates the development of AP [22]. CRP, which can enhance inflammation in endothelial cells [23], is an important factor in predicting the severity and outcome of AP. In our study, there was a downward tendency of the CRP level in the experimental group after treatment. IL-6 and TNF-*α*, which are also expected to be closely related [24], can both influence the severity of AP and be toxic to human cells [23, 25] as well as playing an important role in regulating CRP and predicting the outcome of AP. Our findings revealed that red peony root could significantly decrease the white blood cell count as well as IL-6 and TNF-*α*, consistent with a previous study [21], providing strong evidence for the anti-inflammatory activity of red peony root.

Abnormal pancreatic microcirculation is one of the most important pathologies in AP [26]. Therefore, the anti-inflammatory activity of red peony root can improve pancreatic microcirculation. Recent studies have reported that red peony root can modulate inflammation and alleviate cerebral hypoxia [13, 27, 28], which can also decrease the blood viscosity, inhibit the platelet aggregation, and prolong the prothrombin time [12, 29]. Thus, it is hypothesized that one of the molecular mechanisms of red peony root in treating AP is possibly the improvement of pancreatic microcirculation. However, the specific signaling transduction pathways related to red peony root remain unknown and need to be further investigated in the future. Once identified, they may provide a new therapeutic target for treating various human disorders.

In our study, we were not able to detect the pancreatic microcirculation, but the pancreatic function evidenced by the downward tendency in amylase level might be an indirect predictor that can evaluate the blood supply to the pancreas. In AP patients, a translocation of gut microbiota is commonly observed [30], which can cause disruption of the gut barrier; in addition, the patients can manifest nonspecific symptoms such as abdominal pain, distension, and constipation [31]. Based on our observations, the coloclyster of red peony root efficiently shortened the remission times of abdominal pain and distension and hastened the resumption of self-defecation, supporting the hypothesis that this treatment could enhance gastrointestinal function, possibly by preventing the translocation of gut microbiota. The rebuilding of the normal gut barrier will reduce the release of toxins produced by intestinal pathogenic bacteria into the blood circulation, thus minimizing the risk of toxemia and sepsis.

Our study ended in March 2015. The article was first launched in February 2016 and was in the process of modification and improvement in recent years. The treatment of AP with traditional Chinese medicine is mainly based on compound prescription. The use of single medicine in our study makes drug ingredients more explicit, which is conducive to further development of basic and clinical research. Also, we used coloclysis instead of gavage in our study, which is more advantageous and perspective in the treatment of AP. However, there still were some limitations in our study. First, we only included 60 patients, and all of the patients were from a single center. A large-scale multicenter clinical trial should be conducted in the future as the communication systems among different institutions are updated, which could provide more reliable results. Second, our study did not explore the specific molecular mechanism of red peony root in alleviating AP, which should be further studied in order to completely clarify the role of this traditional Chinese medicine in the treatment of AP.

## 5. Conclusions

The results of our study showed that a coloclyster of red peony root significantly shortened the remission time of the clinical symptoms of MSAP patients as well as the normalization of white blood cells and the percentages of neutrophils and lymphocytes. Moreover, it decreased the modified Balthazar CT score as well as the serum IL-6 and TNF-*α* levels to a greater extent. In summary, a coloclyster of red peony root granules significantly enhanced the therapeutic efficacy of standard treatment and improved the clinical outcomes of our cohort of MSAP patients, possibly by attenuating the inflammatory response, improving pancreatic microcirculation, restoring the gut barrier, and reducing pancreatic autodigestion in a comprehensive manner. Considering the high complication rates and poor outcomes of MSAP patients, our findings could be of valuable clinical significance in guiding the clinical management of such patients. However, this conclusion should be further verified in large-scale clinical trials, which may provide more sound evidence, before the introduction of this treatment into routine clinical practice.

## Figures and Tables

**Table 1:** Observation recording form.

No.	Admisson	Day 1		Day 2		Day 3		Day 4		Day 5		Day 6		Day 7		Discharge
		6:30 AM	4:30 PM	6:30 AM	4:30 PM	6:30 AM	4:30 PM	6:30 AM	4:30 PM	6:30 AM	4:30 PM	6:30 AM	4:30 PM	6:30 AM	4:30 PM	
*Clinical symptoms*																
Fever																
Abdominal pain																
Self-defecation																
Adverse events																
*Laboratory tests*																
White blood cells (×10^9^)																
Neutrophils percentage (%)																
Lymphocytes percentage (%)																
Serum amylase (U/L)																
CRP (mg/L)																
IL-6 (pg/mL)																
TNF-*α* (pg/mL)																
*Radiological examination*																
Modified Balthazar CT score		—	—	—	—	—	—	—	—	—	—	—	—	—	—	

**Table 2:** Compounds identified in positive ion mode and negative ion mode.

No	Name	Rt (min)	Formula	Exp *m*/*z*	[M + X]
1	2-Methoxy-4-vinylphenol	0.752	C_9_H_10_O_2_	151.0752	[M + H]^+^
2	2-Furan formaldehyde	0.776	C_5_H_4_O_2_	97.0285	[M + H]^+^
3	Desbenzoylpaeoniflorin isomer II	1.104	C_16_H_24_O_10_	421.1365	[M + COOH]^−^
4	Glucopyranosyl-paeonisuffrone	1.774	C_16_H_24_O_9_	383.1314	[M + Na]^+^
5	Gallic acid	1.856	C_7_H_6_O_5_	171.0288	[M + H]^+^
6	4-O-Methyldesbenzoylpaeoniflorin	4.606	C_17_H_26_O_10_	389.1466	[M − H]^−^
7	Mudanpioside F	4.778	C_16_H_24_O_8_	343.1415	[M − H]^−^
8	Eugenol	5.334	C_10_H_12_O_2_	165.0908	[M + H]^+^
9	Oxidize paeoniflorin	6.446	C_23_H_28_O_12_	519.1478	[M + Na]^+^
10	Catechin	6.504	C_15_H_14_O_6_	291.0869	[M + H]^+^
11	Paeonol	7.518	C_9_H_10_O_3_	167.0698	[M + H]^+^
12	1-(2-Hydroxy-4-methoxyphenyl)-ethanone	7.641	C_9_H_10_O_3_	167.0701	[M + H]^+^
13	Oxypaeoniflorin	8.05	C_23_H_28_O_11_	481.1708	[M + H]^+^
14	Paeoniflorin	8.189	C_23_H_28_O_11_	481.17	[M + H]^+^
15	4-(1-Methylethenyl)-1-cyclohexene-1-methanol	8.304	C_10_H_16_O	153.1269	[M + H]^+^
16	4-(1-Methylethenyl)-1-cyclohexene-1-carboxaldehyde	8.5	C_10_H_16_O	153.1269	[M + H]^+^
17	Benzaldehyde	8.819	C_7_H_6_O	107.0849	[M + H]^+^
18	1-(2-Hydroxyl-4-methoxyphenyl)-ethyl ketone	9.425	C_9_H_10_O_3_	167.0699	[M + H]^+^
19	Salicylaldehyde	10.57	C_7_H_6_O_2_	123.0441	[M + H]^+^
20	2-Hydroxybenzaldehyde	10.766	C_7_H_6_O_2_	123.0441	[M + H]^+^
21	Gallic acid paeoniflorin	10.898	C_30_H_32_O_15_	631.1681	[M − H]^−^
22	1,2,3,4,6-pentagallic acid glucose	11.372	C_41_H_32_O_26_	939.1105	[M − H]^−^
23	4,7-Dimethyl-benzofuran	11.388	C_10_H_10_O	147.0804	[M + H]^+^
24	1,8-Cineole-2- O-*β*-d-glucopyranoside	12.018	C_16_H_28_O_7_	355.1736	[M + Na]^+^
25	1-Methyl-4-(1-methylethyl)-1,4-cyclohexadiene	15.446	C_10_H_16_	137.1324	[M + H]^+^
26	D-Limonene	15.618	C_10_H_16_	137.1321	[M + H]^+^
27	3,7-Dimethyl ellagic acid	15.864	C_16_H_10_O_8_	331.0458	[M + H]^+^
28	Benzoyl paeoniflorin	16.028	C_30_H_32_O_12_	583.1825	[M − H]^−^
29	Dihydroapigenin	16.166	C_15_H_12_O_5_	273.0764	[M + H]^+^
30	Pentadecanoic acid	16.355	C_15_H_30_O_2_	287.2237	[M + COOH]^−^
31	1-(2-Hydroxy-4-methoxyphenyl)-ethanone	16.379	C_9_H_10_O_3_	167.07	[M + H]^+^
32	Phthalate methyl ethyl ester	16.903	C_12_H_14_O_4_	245.0778	[M + Na]^+^
33	9-Hexadecenoic acid methyl ester	17.075	C_17_H_32_O_2_	313.2388	[M + COOH]^−^
34	(E)-2-Methoxy-5-(1-propenyl)-phenol	17.566	C_10_H_12_O_2_	165.0911	[M + H]^+^
35	Dibutyl phthalate	17.836	C_16_H_22_O_4_	279.1595	[M + H]^+^
36	(Z)-0-Alkene-stearamide	18.703	C_18_H_35_NO	282.2794	[M + H]^+^

**Table 3:** Demographic and clinical data (ITT population).

	Experimental group (*n* = 30)	Control group (*n* = 30)	*p* value
Sex (male/female)	16/14	18/12	0.795
Age	49.5 ± 13.6	47.2 ± 13.6	0.515
Body mass index	23.6 ± 3.2	23.8 ± 3.4	0.887
Etiology			0.710
Biliary diseases	15 (50%)	11 (36.7%)	
Alcohol	10 (33.3%)	11 (36.7%)	
High fat	3 (10%)	4 (13.3%)	
Others	2 (6.7%)	4 (13.3%)	
APACHE II score	9.1 ± 2.2	8.4 ± 2.3	0.207
Modified Balthazar CT score	4.1 ± 0.4	4.3 ± 0.7	0.167

Data are expressed as the mean ± standard deviation. Biliary diseases: chronic history of biliary stones or inflammation. Alcohol: mass drinking history 24–48 h before onset. High fat: triglyceride value was higher than 11.3 or between 5.63 and 11.3, but the serum was milky.

**Table 4:** Comparison of IL-6 and TNF-*α* levels between the two groups (ITT population).

	At admission	At discharge	Differential value
IL-6, pg/mL			
Experimental group (*n* = 30)	91.5 ± 22.8	58.6 ± 18.2^ΔΔ^	33.0 ± 15.0^*∗*^
Control group (*n* = 30)	80.0 ± 25.1	64.7 ± 21.5	15.3 ± 20.4

TNF-*α*, pg/mL			
Experimental group (*n* = 30)	435.8 ± 92.7	329.2 ± 69.7^ΔΔ^	106.7 ± 72.5^*∗*^
Control group (*n* = 30)	377.0 ± 125.3	333.7 ± 91.5	43.3 ± 83.6

Data are expressed as the mean ± standard deviation. Experimental group vs. control group, ^*∗*^*p* < 0.05. Level before treatment vs. level after treatment within one group, ^Δ^*p* < 0.05, ^ΔΔ^*p* < 0.01.

**Table 5:** Comparison of the modified Balthazar CT score between the two groups (ITT population).

Group	At admission	At discharge	Differential value
Experimental group (*n* = 30)	4.1 ± 0.4	2.1 ± 0.5^ΔΔ^^*∗∗*^	1.9 ± 0.4^*∗*^
Control group (*n* = 30)	4.3 ± 0.7	2.9 ± 1.0^ΔΔ^	1.4 ± 1.2

Data are expressed as the mean ± standard deviation. Experimental group vs. control group, ^*∗*^*p* < 0.05, ^*∗∗*^*p* < 0.01. Level before treatment vs. level after treatment within one group, ^Δ^*p* < 0.05, ^ΔΔ^*p* < 0.01.

**Table 6:** Comparison of the duration and cost of hospitalization between the two groups (ITT population).

Group	Duration of hospitalization	Cost of hospitalization
Experimental group (*n* = 30)	11.3 ± 2.3^*∗∗*^	4.15 ± 0.64^*∗∗*^
Control group (*n* = 30)	13.8 ± 2.7	4.84 ± 0.88

Data are expressed as the mean ± standard deviation. Experimental group vs. control group, ^*∗*^*p* < 0.05, ^*∗∗*^*p* < 0.01.

## Data Availability

The data used to support the findings of this study are included within the article and the supplementary information file.

## Abbreviations

AP: Acute pancreatitis
SAP: Severe acute pancreatitis
MSAP: Moderately severe acute pancreatitis
APACHE II: Acute physiology and chronic health evaluation II
BISAP: Bedside Index for Severity In Acute Pancreatitis
MCTSI: Modified Computed Tomography Severity Index
ESI: Electrospray ionization
IL-6: Interleukin-6
TNF-*α*: Tumor necrosis factor-alphaCRP, C-reactive protein
CT: Computed tomography
CI: Confidence interval
ITT: Intention-to-treat
PP: Per-protocol
TICs: Total ion chromatograms.

## Supplementary Materials

Supplementary Materials The more detailed trial design is provided as supplementary material, including the description of the drug, interventions, outcome measurements, randomization and blinding, safety considerations, data management and statistical analysis, and expected outcomes of the study. For the more detailed trial design of the study, refer to
