# Supplementary material for: Coloclyster of Red Peony Root Granules Alleviates Moderately Severe Acute Pancreatitis: A Double-Blinded, Placebo-Controlled, Randomized Clinical Trial
Source: Evid Based Complement Alternat Med. 2020 Jul 22;2020:8401239. doi: 10.1155/2020/8401239 (PMC7396111; doi:10.1155/2020/8401239)
Supplement: Supplementary Materials — The more detailed trial design is provided as supplementary material, including the description of the drug, interventions, outcome measurements, randomization and blinding, safety considerations, data management and statistical analysis, and expected outcomes of the study. For the more detailed trial design of the study, refer to [file 8401239.f1.pdf]

Study goals and objectives

The primary objective of the study was to evaluate optimal efficiency of coloclyster of red peony root to routine treatment in patients with MASP. As a secondary objective, patients' systematic inflammation was also assessed.

Study Design

This was a randomized, double-blind study comprising of a screening period and doubled-blind treatment period. The sample size and power calculations were based on superiority test. Sample size was calculated based on α=0.05 and (1-β)=80%, as previous paper described. The lost of follow-up rate was supposed to be 10%, and the final sample of 30 in each group was determined.

Patients aged 18-70 years old and diagnosed with MSAP in accordance with the global initiative for MASP in Department of Gastroenterology and Emergency of Changhai Hospital from June 2014 to March 2015 were eligible for this study. And the key exclusion criteria included disease course lasting more than 72 h upon admission, pregnancy, the presence of a previous history of AP, severe primary comorbidities including cardiopulmonary or hematological disorders, shock, disseminated intravascular coagulation, acute respiratory distress syndrome, being treated by Chinese herbs before admission, enrollment in other clinical trial. The expected duration of the study was 7 days.

Methodology

Description of the drug

Preparation of sample solutions

The red peony root was purchased from Jiangyin Tianjiang Pharmaceutical Company (Jiangsu, China) and were identified by Professor Hailiang Xin (Navy Military Medical University, Shanghai, China). Granular samples of 0.05 g was accurately weighted into a 50 mL volumetric flask and sonicated with 70% alcohol using an ultrasonicator for 30 min at room temperature. Then, 1.0 mL extraction was transferred to a 1.5 mL microcentrifuge tube and centrifuged for 10 min at 13,000 rpm. The supernatant was finally collected and stored at 4 ℃ pending for analysis.

HPLC–MS analysis

The experiment was performed using an Agilent 1290 Infinity equipped with an Agilent 6538 UHD and Accurate-Mass Q-TOF LC/MS. Samples (4μL) were separated on a Waters XBridge HSS T3 column（2.1mm×100mm, i.d. 2.5um）column using gradient elution. The mobile phase consisted of two solvents: solvent A was 0.1% formic acid; and solvent B was a mixture of 0.1% formic acid in acetonitrile. Gradient elution was performed as follows: 0–2 min, 5% B; 2–10 min, 5–20% B; 10-14 min, 20% B; 14–17 min, 20–95% B; 17–20 min, 95% B． The flow rate was 0.4 mL/min, and column temperature was 25 ℃. The MS identification was operated using an electrospray ionization (ESI) source in positive and negative ion

modes. The ionization source conditions (positive/negative ion mode) were as follows: nebulizer pressure of 50 psig; drying gas flow rate of 11 L/min; drying gas temperature of 350 ℃; capillary voltage of 4.0 kV/-3.5 kV; and fragmentor voltage of 120 V. The spectrometric (MS) data were collected from m/z 500 to 1500 Da in positive and negative ion modes, and stored in centroid mode.

Data analysis and the results

The probable chemical constituents of the red peony root were collected according to related literature, which previously reported constituents in the red peony root and related species. A database of the known chemical constituents was established using the "Formula-Database-Generator" software and then introduced into the "Masshunter Qualitative Analysis" software system provided by Agilent. The identification of the red peony root was carried out by retrieving the MS data of the samples in the database. The molecular ion peaks of $[M+H]^+$, $[M+Na]^+$, $[2M+H]^+$ in positive ion mode and $[M-H]^-$, $[M+HCOO]^-$, $[2M-H]^-$ in negative ion mode were searched. The mass ranges were set at m/z 50-1500 Da, and the mass error of the predicted chemical formula should be less than 5.00 ppm. Finally, a total of 36 compounds were identified in positive ion mode and negative ion mode in the red peony root.

Interventions

A total of 60 MASP patients enrolled in this study were randomly divided into an experimental (n=30) or a control group (n=30). Patients in the experimental or

control group received a coloclyster of 15 g of red peony root or placebo granules dissolved in 150 mL of water at 35-40 ℃ at 9:00 am and 3:00 pm, twice a day for 7 days. In addition, all patients in the two groups had fasted; received gastrointestinal decompression, antacids, and antibiotics; and were supported by parenteral alimentation (A dosage of 20 mg of pantoprazole sodium q12h, 300 mg of gabexate mesylate qd, 300,000 U of ulinastatin qd, 1.5 g of cefoperazone sodium/ sulbactam sodium bid, and 20 μg of alprostadil qd). Early enteral nutrition was also necessary (A dosage of 125 g of Short Peptide Enteral Nutrition Powder qd at the beginning, replaced by 1500-2000 ml of Enteral Nutritional Emulsion qd after 3-5 d).

Outcome measurements

The vital signs, clinical symptoms of abdominal pain and self-defecation, and adverse events were recorded at 6:30 am and 4:30 pm every day in all patients to analyze the therapeutic efficacy of the treatment. The remission time of abdominal pain and self-defecation was the primary outcomes used to determine overall effect. Secondary outcomes were measured at the remission of fever, the modified Balthazar CT score, the remission times for the normalization of white blood cells, percentage of neutrophils and lymphocytes. And the level of serum interleukin-6 (IL-6), tumor necrosis factor-alpha (TNF-α) and hospital stay and cost of hospitalization also were measured or calculated.

Peripheral venous samples were collected from all patients at admission; 6:30 am and 4:30 pm every day during the treatment. Nurses who were responsible for

different patient collected the samples. The serum amylase, C-reactive protein (CRP), IL-6 and TNF-α levels were detected, and routine blood tests were conducted by clinical laboratory of Changhai hospital. Computed tomography (CT) scans were performed at admission and whenever necessary during the treatment. Patients were allowed to be discharged when satisfied all the following conditions, including the disappearance of clinical symptoms, the normalization of blood test, a reduced exudation showed by CT examination, and the formation of pseudocyst. The modified Balthazar CT score was performed by a same person. Adverse events were observed and recorded on all participants through the period by investigator.

Randomization and blinding

An independent statistician generated sixty random numbers using PROC PLAN of SAS 9.2 (SAS Institute Inc., Cary, NC, USA) and kept a sealed copy of the number list. Pharmacy department staff labeled each red peony root granules or placebo with randomized numbers. To maintain the study blinded, there was no discernible differences between the red peony root granules and placebo granules. Those herbs and placebo were subsequently distributed to each investigators. The randomization was controlled by an investigational center, who transmitted the randomization form containing basic information for each enrolled subject by facsimile to the statistician. Then the statistician returned a randomization form filled in with the established random number (specified ID number) to the investigational center. After this process, the investigational center provided the specified ID number to the investigators and

the clinical pharmacists distributed the red peony root granules or placebo granules according to the corresponding number. The randomization allocation ratio to the sites was 1:1. All participants, investigators, investigational center, and pharmacists were blinded to the treatment. All procedures about randomization and blinding were audited by the Ethics Committee of Changhai Hospital.

The key stopping rules for individuals included the occurrence of complications or special physiological changes which were not suitable to continue, poor compliance which could affect the safety and efficacy evaluation, the occurrence of adverse or serious adverse events which were not appropriate for patients to continue, refusal to continue because of personal reasons put forward by patients, and decisions to withdraw made by researchers for other circumstances.

Safety Considerations

The safety of research participants was guaranteed. The informed consents were obtained from all participants. And the adverse events were observed and recorded on all participants by investigator through the period. In the study, all the patients could tolerate the coloclyster procedure, and the success rate was 100%. Few reports of adverse events which associated with the primary disease, the high location, and the rapid procedure of the coloclyster were cured by conservative treatment.

Data Management and Statistical Analysis

All statistical analyses were conducted by SPSS 20.0 software (SPSS Inc., Chicago, IL, USA) by an independent statistician. The differences between the two groups were tested by the independent t-test or the rank-sum test, when applicable. The differences of continuous variables before and after treatment within each group were compared by the paired t-test. A P value less than 0.05 was considered to be statistically significant.

Expected Outcomes of the Study

The coloclyster of red peony root granules could significantly enhance the therapeutic efficacy of standard treatment and improve the clinical outcome of MASP patients. We expected that our findings could be of valuable clinical significance in guiding the clinical management of MASP patients.
